# Robotic vs. conventional total knee arthroplasty over two decades: Evolving trends toward personalised alignment without significant clinical superiority in predominantly mild varus deformity—A systematic review of RCTs

**DOI:** 10.1002/jeo2.70452

**Published:** 2025-12-02

**Authors:** Riccardo Sacco, Andrea Tecame, Matthieu Lalevée, Antoine Perrier, Edward Massa, Pascal Kouyoumdjian, Paolo Adravanti

**Affiliations:** ^1^ Department of Orthopedic and Trauma Surgery Hôpital Charles Nicolle Rouen France; ^2^ CETAPS EA3832, Research Center for Sports and Athletic Activities Transformations University of Rouen Normandy Mont‐Saint‐Aignan France; ^3^ Department of Orthopaedic and Trauma Surgery Città di Parma Clinic Parma Italy; ^4^ Univ. Grenoble Alpes, CNRS, TIMC Grenoble France; ^5^ Groupe Hospitalier Diaconesses–Croix Saint‐Simon Paris France; ^6^ TwInsight Grenoble France; ^7^ Colchester General Hospital Colchester Essex UK; ^8^ Department of Orthopedic and Traumatological Surgery and Spine Surgery Hôpital Universitaire Carémeau Nîmes Cédex 9 France; ^9^ Mechanical and Civil Engineering Laboratory, UMR 5508 CNRS‐UM CC 048 Montpellier 1 University Montpellier France

**Keywords:** randomised controlled trials, robotic‐assisted total knee arthroplasty, robotic surgery, systematic review, total knee replacement

## Abstract

**Purpose:**

Randomised controlled trials (RCTs) comparing robotic‐assisted (RA‐TKA) and conventional total knee arthroplasty (C‐TKA) have demonstrated evolving trends in alignment strategies, surgical workflows and outcomes measurements. This review synthesises current evidence to clarify the evolving role of RA‐TKA over the past two decades.

**Methods:**

A PRISMA systematic review was conducted searching PubMed, Cochrane Library, and Google Scholar (2000–2024). Eligible studies were Level I RCTs comparing RA‐TKA to C‐TKA with well‐defined alignment strategies and surgical workflows. Data included demographics, robotic and prosthetic systems, learning curves, outcome measures and complications.

**Results:**

From 850 records, 9 RCTs were included (627 TKAs: 317 RA‐TKA, 310 C‐TKA). Patients averaged 66.9 years, BMI 28.1 kg/m², 27.9% male, 69.7 knees, follow‐up 23.7 months. Coronal deformity was predominantly varus (<10°). Robotic systems included Robodoc, Mako, and Navio using cruciate‐retaining or posterior‐stabilised prostheses. All earlier studies followed mechanical alignment (MA); after 2021 all RA‐TKAs adopted personalised alignment strategies. Patellar management varied widely, with no RA‐TKA workflow addressing the patellofemoral space. Most procedures were performed by one or two experienced surgeons; learning curves were only noted in early MA studies. RA‐TKA reduced alignment outliers by 10%–24% (*p* < 0.05) versus C‐TKA regardless of the alignment strategy, with no significant difference in functional scores (WOMAC, HSS, KSS, OKS and FJS), range of motion or complications. Recent trials increasingly assessed inflammatory markers, periarticular tissue injury, and quality‐of‐life outcomes.

**Conclusions:**

Over the past two decades, RA‐TKA has evolved substantially, transitioning from purely bone‐cutting technology to decision‐making platforms enabling alignment personalisation and dynamic soft‐tissue assessment, improving surgical precision regardless of alignment strategy. Clinical superiority over C‐TKA remains inconclusive in relatively low‐risk patients, predominantly older females with varus deformity, highlighting the need for studies with more diverse populations, prosthetic design, long‐term follow‐up, patient‐specific outcomes, standardised workflow and learning curve reporting, and patellofemoral space optimisation.

**Level of Evidence:**

Level I, systematic review of non‐homogeneous RCTs.

**Abbreviations**
BMI: body mass index
CR: cruciate retaining
C‐TKA: conventional total knee arthroplasty
FA: functional alignment
FJS: forgotten joint score
HRQoL: health‐related quality of life
KA: kinematic alignment
KSS: Knee Society Score
MA: mechanical alignment
MASTI: multi‐dimensional assessment of surgical trauma impact
OKS: oxford knee score
PS: posterior stabilised
RA‐TKA: robotic‐assisted total knee arthroplasty
RCTs: randomised controlled trials
ROM: range of motion
VAS: visual analogue scale
WOMAC: Western Ontario and McMaster Universities Osteoarthritis Index

## INTRODUCTION

The adoption of robotic‐assisted total knee arthroplasty (RA‐TKA) has risen exponentially in recent years [24, 38], demonstrating improved implant positioning accuracy and reduced iatrogenic injury to periarticular tissues [14, 17, 50]. Projections estimate that RA‐TKA will account for 49.9% of all knee arthroplasties by 2030 [18]. However, randomised controlled trials (RCTs) and meta‐analyses have not demonstrated significant improvements in patient‐reported outcome measures (PROMs), knee range of motion (ROM), or revision rates when compared to conventional, jig‐based total knee arthroplasty (C‐TKA) at short‐ to mid‐term follow‐up [3, 42,]. High‐quality comparative studies are needed to evaluate the long‐term functional outcomes, cost‐effectiveness, and implant survivorship of RA‐TKA compared to C‐TKA [8, 22, 28].

A limitation of the current literature is the heterogeneity in robotic surgical systems and workflows, which are influenced by factors such as surgeon experience, alignment strategy, preoperative planning accuracy, and soft tissue management [22, 38]. Earlier studies comparing RA‐TKA and C‐TKA primarily used robotic assistance for bone cuts, followed by sequential soft tissue release aimed at achieving mechanical alignment (MA) [7, 63]. More recent robotic approaches facilitate alignment personalisation and intraoperative soft tissue assessment, integrating kinematic alignment (KA) and functional alignment (FA) strategies to minimise iatrogenic soft tissue release [9, 11, 30, 41]. Despite these advancements, uncertainty remains regarding the optimal role of robotic assistance in TKA. While robotic systems enhance precision in bone preparation [25, 50], their primary purpose, whether to improve MA accuracy or to enable personalised alignment strategies, remains debated [12, 16, 30]. Robotic assistance facilitates individualised 3D preoperative planning based on the three‐compartment knee phenotype concept [21], aligning prosthetic implantation with the patient's unique 3D bony anatomy in the coronal, sagittal, and axial planes, while considering prosthetic design and native ligamentous tension. However, the extent to which robotic assistance should influence intraoperative decision‐making, particularly in modifying preoperative plans based on intraoperative soft tissue assessment, remains controversial and largely dependent on surgeon preference [23, 58]. Further research is needed to determine whether these theoretical advantages translate into improved clinical outcomes. Additionally, few studies have comprehensively evaluated surgeon expertise with robotic systems beyond implant positioning precision and operative duration [58, 59]. All these variables introduce significant methodological variability and could impact the interpretation of comparative outcomes between RA‐TKA and C‐TKA.

This study critically examines the methodology of RCTs comparing RA‐TKA and C‐TKA to assess whether these key limitations have been addressed. We hypothesise that, over the past two decades, RA‐TKA has shown evolving trends in alignment strategies, surgical workflows, and outcome measurements.

## MATERIALS AND METHODS

This systematic review was carried out following the Preferred Reporting Items for Systematic Reviews (PRISMA) guidelines [46, 48].

### Search strategy

A systematic search was conducted using PubMed, the Cochrane Library, and Google Scholar to identify relevant articles published in peer‐reviewed journals between January 1, 2000, and January 1, 2025. The search strategy included the following keywords and their variations: “Knee Arthroplasty,” “Knee Replacement,” “Joint Replacement,” “Total Knee,” “Robotic Assisted,” “Robotic Surgery,” “Robotic,” “Conventional,” “Manual,” and “Standard Technique” (Supporting Information: Table 1).

Two authors (RS and AT) independently screened the search results for RCTs comparing RA‐TKA with C‐TKA, according to predefined eligibility criteria (Supporting Information: Table 2), based on titles and abstracts. All retrieved articles were imported into EndNote reference management software to facilitate screening and deduplication. Discrepancies were resolved through discussion with a third senior author (PA). Full‐text articles meeting the inclusion criteria were thoroughly reviewed, and reference lists were manually screened to identify any additional relevant studies.

### Data collection and items

Data was extracted using a pre‐designed Microsoft Excel sheet including the surname of the first author, year of study, patient demographics (age, gender and sample size), prosthesis type and robotic system used, length of the follow‐up and primary outcomes, description of robotic surgical workflow, and learning curve. A predefined protocol was established in advance to guide the review process (Supporting Information: PRISMA Protocol S2). The risk‐of‐bias visualisation was generated using the RoB 2.0 tool with the ROBVIS application (https://mcguinlu.shinyapps.io/robvis/).

### Statistical analysis and outcomes of interest

This systematic review employed a qualitative synthesis of RCTs comparing RA‐TKA with C‐TKA, without conducting a pooled quantitative meta‐analysis due to significant heterogeneity in surgical workflows, alignment strategies, and outcome measures. Outcomes of interest included radiographic alignment, postoperative ROM, functional scores (Western Ontario and McMaster Universities Osteoarthritis Index [WOMAC], Knee Society Score [KSS], Oxford Knee Score [OKS] and Hospital for Special Surgery Knee Score [HSS]), inflammatory markers, periarticular soft tissue injury, and quality of life metrics (e.g., SF‐36), as well as the learning curve associated with RA‐TKA. Descriptive statistics, including means, standard deviations, and ranges, were employed to summarise demographic characteristics and outcomes. Data were analysed in the context of the chronological evolution of RA‐TKA over the past two decades, with attention to technological advancements, changes in surgical workflows, and alignment strategies.

## RESULTS

**Figure 1:**
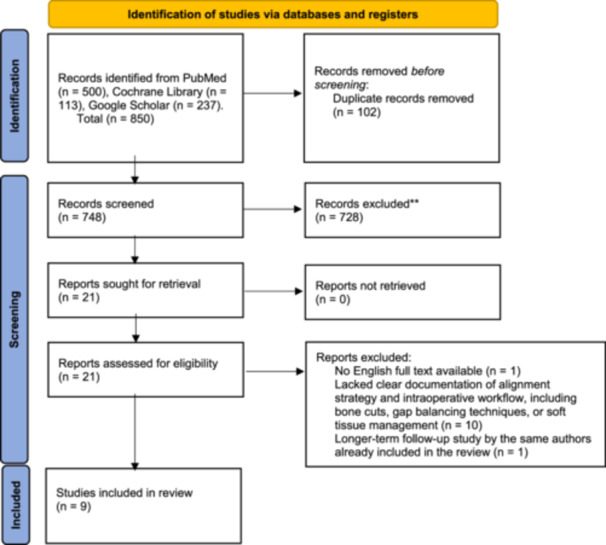
Flow diagram of the screening and selection process. A total of 850 records were identified across three databases: PubMed (500), Cochrane Library (113) and Google Scholar (237). After removing 102 duplicate entries, 748 unique records were screened based on titles and abstracts. Of these, 728 were excluded for not meeting the criteria for randomised controlled trials (RCTs), leaving 21 RCTs for full‐text review. During the eligibility assessment, 12 RCTs were excluded for various reasons: one study was not published with a full English text; [1] 10 lacked clear documentation of alignment strategy and intraoperative workflow, including bone cuts, gap balancing techniques, or soft tissue management; [2, 28, 32, 35, 37, 55, 57, 62, 63, 64] and one was excluded because a longer‐term follow‐up study by the same authors was already included in the review [34]. The remaining nine RCTs, which demonstrated a clear and reproducible intraoperative surgical workflow, were included in the systematic review [8, 13, 19, 26, 33, 47, 49, 51, 54]. *Consider, if feasible to do so, reporting the number of records identified from each database or register searched (rather than the total number across all databases/registers). **If automation tools were used, indicate how many records were excluded by a human and how many were excluded by automation tools. *Source*: Page et al. [46]. This work is licensed under CC BY 4.0. To view a copy of this license, visit https://creativecommons.org/licenses/by/4.0/.

The study selection process is detailed in Figure 1, illustrating the identification, screening, and inclusion of nine RCTs based on predefined eligibility criteria. After removing 102 duplicate entries, 748 unique records were screened based on titles and abstracts. Of these, 728 were excluded for not meeting the criteria for RCTs, leaving 21 RCTs for full‐text review. During the eligibility assessment, 12 RCTs were excluded for various reasons: one study was not published with a full English text; [1] ten lacked clear documentation of alignment strategy and intraoperative workflow, including bone cuts, gap balancing techniques, or soft tissue management [2, 28, 32, 35, 37, 53, 56, 62, 63, 64], and one was excluded because a longer‐term follow‐up study by the same authors was already included in the review [34]. The remaining 9 RCTs, which demonstrated a clear and reproducible intraoperative surgical workflow, were included in the systematic review [8, 13, 19, 26, 33, 47, 49, 51, 54]. An overview of RCTs comparing RA‐TKA and C‐TKA is shown in Table 1.

**Table 1:** Overview of RCTs comparing robotic‐assisted (RA‐TKA) and conventional total knee arthroplasty (C‐TKA) summarising patient demographics, follow‐up duration, prosthesis and robotic systems used, and primary outcomes.

Study and year of publication	Age (M ± SD)	Sex (male, *n*%)	BMI (kg/m²)	RA‐TKA study arm	C‐TKA study arm	Follow‐up	Primary outcomes	Type of prosthesis	Robot system	Conclusion
Park and Lee 2007 [47]	62.7 ± 6.51 (RA‐TKA), 67.8 ± 6.44 (C‐TKA)	NR	NR	32	30	48 months	KSS, loosening, prosthetic alignment, complications	PS LPS Zimmer	ROBODOC	RA‐TKA improved planning and alignment but had higher early complications (fractures, infections, nerve injuries).
Song et al. 2011 [49]	67 ± 6.3 (RA‐TKA and C‐TKA)	0 (0%)	27 ± 6.5	30	30	12 months	Percentage of cases within ± 3° of neutral alignment in bilateral TKA	CR NexGen Zimmer	ROBODOC	RA‐TKA had less bleeding, fewer outliers, but required longer incisions and operative time, with similar clinical outcomes.
Song et al. 2013 [51]	66.1 ± 7.1 (RA‐TKA), 64.8 ± 5.3 (C‐TKA)	4 (8%) (RA‐TKA), 5 (10%) (C‐TKA)	26.3 ± 2.7 (RA‐TKA), 26.2 ± 3.9 (C‐TKA)	50	50	41 months	Percentage of cases within ± 3° of neutral alignment	CR NexGen Zimmer	ROBODOC	RA‐TKA improved alignment and gap balance, but showed similar clinical outcomes and complications.
Liow et al. 2017 [33]	NR	NR	NR	31	29	2 years	ROM, OKS, KSS, SF‐36, Health Survey scores	PS LPS Zimmer	ROBODOC	RA‐TKA showed slight enhancements in quality of life measures compared to conventional TKA.
Thiengwittayaporn et al. 2021 [54]	69.0 ± 8.3 (RA‐TKA), 69.1 ± 7.3 (C‐TKA)	6 (8%) (RA‐TKA), 15 (19.5%) (C‐TKA)	28.0 ± 4.9 (RA‐TKA), 27.7 ± 4.6 (C‐TKA)	75	77	1.5 months (6 weeks)	Implant positioning accuracy, alignment of mechanical axis, femoral tibial inclinations, joint line changes	PS Legion Smith & Nephew	NAVIO (Imageless)	RA‐TKA showed superior alignment, fewer outliers, and minimal joint line changes with a short learning curve.
Kayani et al. 2021 [26]	67.9 ± 8.6 (RA‐TKA), 68.7 ± 9.6 (C‐TKA)	6 (40%) (RA‐TKA), 7 (47%) (C‐TKA)	27.0 ± 3.0 (RA‐TKA), 27.5 ± 3.7 (C‐TKA)	15	15	28 days	Inflammatory markers (IL‐6, TNF‐α, CRP), MASTI scores, alignment accuracy	CR Triathlon Stryker	Mako	RA‐TKA reduced early postoperative inflammation, minimised soft tissue and bone trauma, and improved component positioning accuracy, with no long‐term inflammatory differences.
Fontalis et al. 2022 [19]	67.9 ± 8.6 (RA‐TKA), 68.7 ± 9.6 (C‐TKA)	6 (40%) (RA‐TKA), 7 (47%) (C‐TKA)	27.0 ± 3.0 (RA‐TKA), 27.5 ± 3.7 (C‐TKA)	15	15	2 years	Local inflammatory markers (IL‐6, IL‐8, TNF‐alpha), pain, opiate requirements, ROM, time to discharge	CR Triathlon Stryker	Mako	RA‐TKA reduced early inflammation and pain, but 2‐year PROMs and quality of life were comparable.
Bollars et al. 2023 [8]	64.4 ± 8.7 (RA‐TKA), 66.4 ± 7.2 (C‐TKA)	11 (42.3%) (RA‐TKA), 9 (34.6%) (C‐TKA)	28.7 ± 4.4 (RA‐TKA), 28.7 ± 4.7 (C‐TKA)	26	26	1.5 months (6 weeks)	Implant placement accuracy by comparing preoperative and postoperative CT scans	PS Journey II Smith & Nephew	NAVIO (Imageless)	RA‐TKA enabled precise implant positioning and significantly reduced outliers, aiming for constitutional alignment.
Clement et al. 2024 [13]	67.0 ± 8.6 (RA‐TKA), 66.5 ± 8.6 (C‐TKA)	22 (51.2%) (RA‐TKA), 16 (42.1%) (C‐TKA)	31.2 ± 5.4 (RA‐TKA), 31.5 ± 7.0 (C‐TKA)	43	38	12 months	Knee‐specific measures (WOMAC, OKS, FJS), HRQoL (EQ‐5D, EQ‐VAS)	CR Triathlon Stryker	Mako	RA‐TKA improved pain relief and met expectations within 12 months, but no significant difference in function or HRQoL.

Abbreviations: BMI, body mass index; CR, cruciate retaining; FJS, forgotten joint score; HRQoL, health‐related quality of life; KSS, Knee Society Score; MASTI, multi‐dimensional assessment of surgical trauma impact; OKS, oxford knee score; PS, posterior stabilised; RCTs, randomised controlled trials; VAS, visual analogue scale; WOMAC, Western Ontario and McMaster Universities Osteoarthritis Index.

### Quality assessment

**Figure 2:**
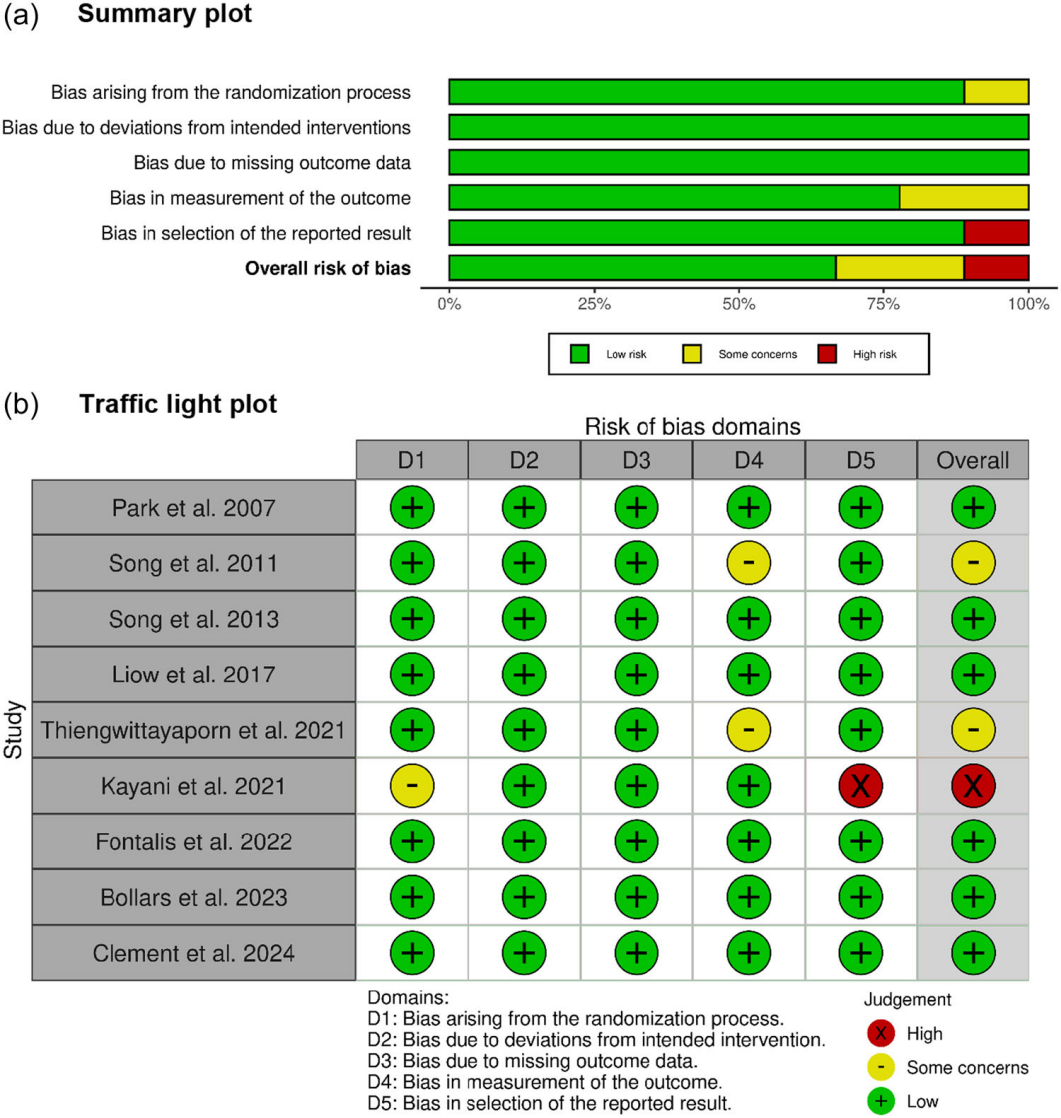
The risk‐of‐bias visualisation was generated using the RoB 2.0 tool with the ROBVIS application (https://mcguinlu.shinyapps.io/robvis/). (a) summary plot and (b) traffic light plot across five bias domains. The visualisation indicates an overall low risk of bias among the studies.

The nine included RCTs demonstrated overall a low risk of bias across five domains, as illustrated in Figure 2.

### General characteristics

Overall, 627 TKAs were included, C‐TKA was performed in 310 knees (49.4%) and RA‐TKA in 317 knees (50.6%); mean total number of knees per study 69.7 ± 38.0 (RA‐TKA: 35.2 ± 18.8; C‐TKA: 34.4 ± 19.2) (Table 2). The mean patient age was 66.9 ± 7.7 years, BMI 28.1 ± 4.3 kg/m², 27.9% males (range 0%–51.2%). Average follow‐up 23.7 months (range 1–48).

**Table 2:** General characteristics of included studies.

Variable	RA‐TKA group	C‐TKA group	Total
Number of TKAs	317	310	627
Mean knees per study	35.2 ± 18.8	34.4 ± 19.2	69.7 ± 38.0
Median knees per study	31.0	30.0	30.0
Male proportion (%)	27.1% (0%– 51.2%)	28.6% (0%– 47%)	27.9% (0%–51.2%)
Mean age (years)	66.5 ± 7.8	67.4 ± 7.5	66.9 ± 7.7
Mean BMI (kg/m²)	28 ± 3.9	28.2 ± 4.8	28.1 ± 4.3
Mean follow‐up (months)	—	—	23.7 ± 21.9
Median follow‐up (months)	—	—	12

Abbreviations: BMI, body mass index; C‐TKA, conventional total knee arthroplasty; RA‐TKA, robotic‐assisted total knee arthroplasty.

### Outcomes

#### Robotic and prosthetic systems, surgical approach

Various robotic systems, imageless [8, 54] or based on preoperative CT scans [13, 19, 26, 33, 47, 49, 51] have been used (Table 1). The prostheses used comprised only two different designs: posterior stabilised (PS) [8, 33, 47, 54] and cruciate retaining (CR) [13, 19, 26, 49, 51]. The ROBODOC system (Integrated Surgical Systems) was employed in four studies (44.5%, 4/9) [33, 47, 49, 51] with the Zimmer cruciate retaining NexGen and LPS prostheses. The Mako system (Stryker) was used in three studies (33.3%, 3/9) [13, 19, 26] with the Stryker Triathlon cruciate‐retaining prosthesis. The NAVIO system (Smith & Nephew) was used in two studies (22.2%, 2/9) with posterior stabilised implants [8, 54], in one of them with 3° varus obliquity in the polyethylene [8]. A medial parapatellar approach was reported in six out of nine studies [8, 19, 26, 33, 49, 51]. The included studies varied in their surgical management of the patella, which was reported in six of the nine studies [8, 13, 19, 26, 54]. Patellar resurfacing was performed in three studies [8, 19, 26], with asymmetrical components used in two of them [19, 26]. Patelloplasty was performed in one trial [33]. In one study, the need for patellar resurfacing was an exclusion criterion for patient selection [13].

### Alignment outliers

Among the four studies that reported alignment outlier rates [8, 49, 51, 54], RA‐TKA consistently demonstrated significantly fewer outliers than C‐TKA regardless of the alignment strategy (Table 3). This corresponds to a 10%–24% reduction in alignment outliers with RA‐TKA, as assessed by postoperative full‐length lower limb radiographs or CT scans. While CT‐based systems like ROBODOC [49, 51] demonstrated complete elimination of mechanical axis outliers, defined as a deviation > ±3° from neutral, imageless systems such as NAVIO [8, 54] also showed significant but less pronounced reductions with a robotic outlier rate ranging from 5.3% to 5.8%. Outlier reduction of RA‐TKA compared to C‐TKA was approximately 23.3%–24% for image‐based systems compared to 10.3%–18.6% for imageless systems. These findings suggest that although both approaches improve alignment over conventional techniques, CT‐based robotic systems may offer greater precision.

**Table 3:** Comparison of alignment outliers, defined as deviation > ±3° from neutral, between C‐TKA and RA‐TKA.

Authors and robotic system	C‐TKA	RA‐TKA	Outliers (C ‐TKA vs. RA‐TKA) (%)	*p* value	Reduction in outliers (C‑TKA vs. RA‑TKA) (%)
Song et al. [49] (ROBODOC, preoperative CT scan)	30	30	23.3% (C) ‐ 0% (RA)	0.001	23.3%
Song et al. [51] (ROBODOC, preoperative CT scan)	50	50	24% (C) ‐ 0% (RA)	<0.001	24.0%
Thiengwittayaporn et al. [54](NAVIO, imageless)	77	75	15.6% (C) ‐ 5.3% (RA)	0.035	10.3%
Bollars et al. [8] (NAVIO, imageless)	26	26	24.4% (C) ‐ 5.8% (RA)	<0.001	18.6%

Abbreviations: C‐TKA, conventional total knee arthroplasty; RA‐TKA, robotic‐assisted total knee arthroplasty.

### An overview of functional outcome scores comparing C‐TKA and RA‐TKA

#### WOMAC

Two studies reported WOMAC scores at 1 year [13, 49], and two others at 2 years of follow‐up [19, 51]. Overall, RA‑TKA resulted in slightly better WOMAC scores across all studies; however, these differences did not reach statistical significance. The observed improvements remained below the Minimal Clinically Important Difference (MCID) for the WOMAC score. In the study by Clement et al. [13], although the total WOMAC score difference was not statistically significant, the WOMAC pain subscale showed a significant improvement in the RA‐TKA group (*p* = 0.029) (Table 4).

**Table 4:** Functional outcome scores (WOMAC, OKS, KSS and ROM) comparing C‐TKA and RA‐TKA.

	Preoperative (C‐TKA)	Preoperative (RA‐TKA)	*p* value	Postoperative (C‐TKA)	Postoperative (RA‐TKA)	*p* value	Mean difference (95% CI)	Follow‐up
**WOMAC**								
**Authors group**								
Song et al. [49]	75 ± 15.0	80 ± 16.0	ns	20.1 ± 8.5	18.5 ± 4.0	ns	nr	1 year
Song et al. [51]	75.2 ± 11.1	65.6 ± 10.2	ns	30 ± 7.5	28.9 ± 4.4	ns	nr	2 years
Fontalis et al. [19]	67.6 ± 12.6	68.7 ± 13.4	0.824	6.13 ± 5.12	4.86 ± 5.81	0.532	nr	2 years
Clement et al. [13]	42.3 ± 18.7	39.6 ± 15.1	0.469	37.6 ± 19.7	41.0 ± 19.3	0.437	3.4 (−5.3 to 12.1)	1 year
**OKS**								
**Study**								
Liow et al. [33]	38.2 ± 9.5	33.6 ± 7.8	ns	17.7 ± 4.2	18.3 ± 7.0	ns	0.6 [−2.5 to 3.7]	2 years
Fontalis et al. [19]	18.20 ± 4.73	18.13 ± 7.18	0.976	45.20 ± 2.75	44.93 ± 2.78	0.794	nr	2 years
Clement et al. [13]	19.6 ± 7.3	19.5 ± 7.7	0.970	20.2 ± 9.6	19.7 ± 10.0	0.814	−0.5 (−4.9 to 3.8)	1 year
**KSS – Knee Score**								
**Study**								
Park and Lee [47]	nr	nr	ns	90.9 ± 4.88	91.6 ± 2.94	ns	nr	2 years
Liow et al. [33]	34.0 ± 17.1	34.3 ± 14.6	ns	87.9 ± 10.6	81.8 ± 14.9	ns	6.1 [−13.1 to 0.9]	2 years
**ROM**								
**Study**								
Park and Lee [47]	nr	nr	nr	122 ± 16.9	118 ± 9.0	ns	nr	2 years
Song et al. [49]	123 ± 14.3	120 ± 16.0	ns	129 ± 12.8	129 ± 13.8	ns	nr	1 year
Song et al. [51]	123 ± 12.3	125 ± 7.6	ns	129 ± 12.4	128 ± 5.1	ns	nr	2 years
Liow et al. [33]	119.8 ± 17.9	121.0 ± 17.4	ns	125.2 ± 10.3	118.3 ± 15.6	ns	0.2 [−2.2 to 1.8]	2 years

*Note*: Values were compared between preoperative and postoperative assessments and are expressed as mean ± standard deviation, and as mean differences with 95% confidence intervals (CI). It is important to highlight that all studies included in the table utilised either the ROBODOC or Mako systems, with no imageless robotic trials represented in this comparison.

Abbreviations: C‐TKA, conventional total knee arthroplasty; KSS, Knee Society Score; OKS, Oxford Knee Score; RA‐TKA, robotic‐assisted total knee arthroplasty; ROM, range of motion; WOMAC, Western Ontario and McMaster Universities Osteoarthritis Index.

### OKS

Among both one and two years follow‐up periods [13, 19, 33], no statistically significant differences in postoperative OKS were observed between RA‐TKA and C‐TKA groups, regardless of alignment strategy. Studies using both mechanical [33] and functional alignment [13, 19] demonstrated comparable outcomes (Table 4).

#### KSS – Knee score

Two RCTs [33, 47] reported on KSS – Knee Scores. Park and Lee [47] found no significant difference at 2 years, with postoperative scores of 90.9 ± 4.88 for C‐TKA and 91.6 ± 2.94 for RA‐TKA. Similarly, Liow et al. [33] observed comparable functional improvements in both groups over two years. Although baseline scores were nearly identical (34.0 ± 17.1 for C‐TKA vs. 34.3 ± 14.6 for RA‐TKA), postoperative KSS slightly favoured C‐TKA (87.9 ± 10.6 vs. 81.8 ± 14.9). However, the mean difference of 6.1 points (95% CI, −13.1 to 0.9) was not statistically significant (Table 4).

### HSS score

Only Song et al. [49, 51] reported on HSS scores. Both studies showed that RA‑TKA and C‑TKA yielded similarly high postoperative scores. While the robotic group had a slightly higher average in both articles, the difference, 1–1.5 points, was too small to indicate a clinical advantage based on HSS alone. In the bilateral TKA comparison using MA in both knees, Song et al. [49] reported similar HSS scores at 1‐year follow‐up: 95.9 (SD ± 5.2) for RA‑TKA and 94.7 (SD ± 5.5) for C‑TKA.

### ROM

Four studies assessed ROM [33, 47, 49, 51], consistently reporting comparable outcomes between RA‐TKA and C‐TKA, with no significant differences observed across groups (Table 4).

#### Forgotten Joint Score (FJS), quality‐of‐life (QoL)

According to Liow et al. [33], at 2‐year follow‐up, RA‐TKA patients showed subtle yet statistically significant improvements in SF‐36 quality of life domains, specifically vitality (*p* = 0.03) and role emotional (*p* = 0.02), with a higher proportion achieving clinically meaningful improvement in vitality scores (MCID: 48.4% vs. 13.8%; *p* = 0.009). Clement et al. [13] was the only study to report on the FJS; at 1 year, scores were 6.9 ± 7.1 for RA‐TKA and 7.7 ± 7.8 for C‐TKA, with a mean difference of 0.8 points (95% CI –2.5 to 4.1; *p* = 0.627), indicating no significant advantage of robotic assistance.

#### Surgical workflow and alignment strategies

Studies employing MA strategies [33, 47, 49, 51, 54] used robotic assistance primarily to enhance the precision of bone cuts based on preoperative planning aimed at achieving MA of the lower limb, followed by conventional soft tissue balancing (Table 5). Among these, Song et al. [49, 51] used a manual commercial tensor device for soft tissue assessment. In contrast, studies focusing on personalised alignment strategies [8, 13, 19, 26] used robotic systems that adapted the surgical plan intraoperatively, starting from either a MA [8, 19, 26] or KA [13] reference point to achieve FA. These systems integrated dynamic evaluation of the soft tissue envelope, enabling fine adjustments to bone resections and implant positioning to realise a personalised alignment strategy, with minimal or no need for soft tissue releases. Only Fontalis et al. [19] presented a flow algorithm for a stepwise approach to achieving FA. Notably, none of the included studies specifically addressed patellofemoral space optimisation within the robotic surgical workflow (Table 5).

**Table 5:** Comparison of TKA surgical workflows, alignment strategies and preoperative coronal knee deformity.

Study	Soft tissue balancing approach	Robotic alignment philosophy	Robotic workflow summary	Preoperative coronal knee deformity
**Mechanical alignment studies**				
Park and Lee [47]	Manual “conventional” soft tissue balancing	MA	Robot used for bone cutting based on CT. Manual implantation and conventional soft tissue balancing afterward.	Not reported
Song et al. [49]	Tensioning device used: medial/lateral gap difference < 3 mm in flexion and extension	MA	Robot used for bone cutting after CT‐based planning. Soft tissue release performed post‐cutting with a tensioning device.	Between 20° varus and 5° valgus; mean mechanical axis preoperative 10.9° varus
Song et al. [51]	Commercial tensor device: equal medial and lateral gaps within ± 2 mm	MA	Bone cuts done independently of soft tissues. Balancing performed post‐cutting using a commercial tensor.	Mechanical axis between 20° of varus and 5° of valgus
Liow et al. [33]	Manual “conventional” soft tissue balancing	MA	Virtual MA planning and patient‐specific bone cuts performed by robot. No soft tissue‐specific adaptation during cuts.	Varus deformity and fixed flexion deformity < 15° (mean varus not specified)
Thiengwittayaporn et al.[54]	Valgus/varus stress target < 3 mm of medial/lateral opening	MA	Pre‐cut planning based on manual soft tissue stress, but cuts made without real‐time adjustment; balancing checked afterward.	HKA angle: RA‐TKA 169.1° (SD 6.6), C‐TKA 170.7° (SD 4.8)
**Personalised alignment studies**				
Kayani et al. [26]	Robotic feed‐back of soft tissue balance aiming to symmetrical gaps with minimal releases	FA (MA starting point)	Intraoperative fine‐tuning of preoperative MA plan to achieve functional alignment using robotic evaluation of soft tissue tension.	Preoperative limb alignment: RA‐TKA mean 3.4° varus (SD 0.9), C‐TKA 3.1° varus (SD 0.7)
Fontalis et al. [19]	Robotic feed‐back of soft tissue balance	FA (MA starting point)	Initial neutral MA plan intraoperatively modified to align with the soft tissue envelope for FA, with step‐by‐step approach.	Preoperative limb alignment HKA: RA‐TKA mean 3.4° varus (SD 0.9), C‐TKA 3.1° varus (SD 0.7)
Bollars et al. [8]	Robotic feed‐back of soft tissue balance	FA (MA starting point)	Intraoperative stress tests used to adapt implant positioning to ligament tension, achieving FA based on CPAK.	Preoperative HKA: RA‐TKA mean 3.3° varus (SD 4.8), C‐TKA 1.0° varus (SD 5.2)
Clement et al. [13]	Robotic feed‐back of soft tissue balance	FA (restricted KA starting point)	The femur was cut first following kinematic alignment principles, and the tibial cut was subsequently adjusted using gap balancing to achieve alignment within restricted KA boundaries.	Varus < 20°

*Note*: Mechanical alignment (MA) studies used manual or tensioner‐based soft tissue balancing, with robotic assistance utilised for bone cuts. In contrast, personalised alignment studies employed functional alignment (FA) strategies, incorporating robotic feedback on soft tissue tension to individualise implant positioning with a MA or kinematic alignment (KA) starting point. Preoperative coronal deformity was predominantly mild to moderate varus ( < 10°).

Abbreviations: C‐TKA, conventional total knee arthroplasty; RA‐TKA, robotic‐assisted total knee arthroplasty; SD, standard deviation.

### Preoperative coronal knee deformity

Eight studies reported on preoperative knee deformity [8, 13, 19, 26, 33, 49, 51, 54] (Table 5). Most included patients with mild to moderate varus deformities, with average preoperative limb alignments ranging from 1.0° to 3.4° of varus, indicating mild coronal deformity [8, 19, 26]. One study [54] reported moderate varus deformity, with mean preoperative HKA angles of 170.7° for C‐TKA and 169.1° for RA‐TKA, corresponding to less than 10° of varus. In contrast, two studies [49, 51] included a broader range of deformities, from up to 20° of varus to 5° of valgus, with a mean preoperative mechanical axis deviation of 10.9° varus. Clement et al. [13] also included patients with varus deformities up to 20°. Liow et al. [33] enroled knees with varus alignment combined with fixed flexion deformity < 15°, though the exact mean varus was not specified. Overall, these findings indicate that most included RCTs focused on a relatively homogeneous population with mild to moderate varus deformities [8, 13, 19, 26], while only a few included valgus deformities without specifying their proportion [49, 51] (Table 5).

### Surgeon experience and learning curve

Surgeon experience was reported in six out of nine studies [26, 33, 47, 49, 51, 54]. In four of these, TKAs were performed by a single experienced surgeon [33, 49, 51, 54], while two studies involved two surgeons [26, 47]. Surgeon experience was not reported in two studies [8, 19], and another one involved surgeons from a single center without further detail [13]. The learning curve was addressed in only three studies [47, 49, 54], all comparing TKA performed with MA. Importantly, patients treated during the learning curve period were included in the final analyses [47]. Thiengwittayaporn et al. [55] estimated the learning curve for robotic surgical time normalisation at approximately seven cases. Park et al. [47] reported higher complication rates during the RA‐TKA learning phase. Only one study reported the number of RA‐TKAs performed before trial participation, >150 cases [49], with a follow‐up trial by the same author [51]. Although personalised alignment trials for RA‐TKA [8, 13, 19, 26] referenced prior data on robotic proficiency, none specifically reported learning curves for RA‐TKA.

### Inflammatory markers, macroscopic soft tissue injury (MASTI) classification system

Within the first postoperative week, RA‐TKA demonstrated transient but significant reductions in systemic and local inflammatory markers (IL‐6, IL‐8, TNF‐α, ESR and CRP), decreased periarticular soft tissue and bone trauma, reduced pain scores, and lower opioid consumption (*p* < 0.001) [19, 26]. RA‐TKA was associated with significantly improved preservation of the periarticular soft tissue envelope (*p* < 0.001), and reduced femoral (*p* = 0.012) and tibial (*p* = 0.023) bone trauma compared with C‐TKA [26] according to the MASTI classification system. Song et al. [51] reported significantly less postoperative drainage in the RA‐TKA group (613 ± 318 mL) compared to the conventional group (933 ± 467 mL; *p* < 0.001).

### Operative time and complications

Earlier studies [33, 49, 51] reported longer operative times for RA‐TKA compared to C‐TKA, with an average increase of 25 min [49]. Thiengwittayaporn et al. [54] identified a learning curve inflection after seven cases, with operative times decreasing from 100.7 to 67.4 minutes. More recent data from Kayani et al. [26] showed no significant time difference (*p* = 0.621) between RA‐TKA (62.4 min) and C‐TKA (61.4 min), indicating time convergence with increased surgeon experience and technological refinement. Early RA‐TKA series reported serious complications during the learning curve [33, 47] such as a patellar tendon rupture and a femoral supracondylar fracture, but no major adverse outcomes after learning completion. Notably, Liow et al. [33] reported a 10% intraoperative abortion rate due to robotic system errors, requiring conversion to conventional techniques in three cases. However, recent studies [13, 26] found no significant differences in local or systemic complications within the first 12 months, suggesting comparable safety profiles between RA‐TKA and C‐TKA. None of the reviewed RCTs reported complications directly related to robotic frame pin placement.

## DISCUSSION

This systematic review of RCTs comparing RA‐TKA with conventional techniques has demonstrated advancements of robotic assistance from MA toward personalised alignment strategies over the past two decades, consistently showing improved alignment accuracy. The data reflect a continuous evolution of RA‐TKA, with early concerns over prolonged operative time and learning‐curve‐related complications progressively addressed through surgeon experience and technological advancements. The reviewed trials predominantly included relatively low‐risk patient populations, composed mostly of females over 60 years of age, presenting primarily with mild to moderate varus knee deformities (<10°) [8, 13, 19, 26, 33, 49, 51, 55] (Tables 2 and 5). While these strict criteria enhance internal validity, they also limit the generalisability of the findings to the broader and heterogeneous patient populations commonly encountered in routine arthroplasty practice, such as patients with significantly different age ranges, obesity or valgus deformities. On the other hand, substantial heterogeneity existed regarding robotic systems (imageless/image‐based), alignment strategies (mechanical/personalised alignment), soft‐tissue balancing techniques (manual, tensor‐based, robotic feedback), and patellar management (resurfacing, patelloplasty or no intervention). Follow‐up was predominantly less than two years, average 23.7 months, limiting insights into mid‐ to long‐term outcomes. Consequently, meta‐analyses comparing RA‐TKA to C‐TKA [3, 7, 42, 43] should be interpreted with caution, as the underlying studies exhibit substantial methodological heterogeneity (Table 1).

A consistent finding was the superior implant positioning accuracy in RA‐TKA, with 10% to 24% fewer outliers (defined as a deviation > ±3° from neutral) compared to C‐TKA, regardless of the alignment strategy (Table 3). This result is supported by existing literature [2, 5, 14]. While CT‐based systems like ROBODOC [49, 51] demonstrated complete elimination of mechanical axis outliers, imageless systems such as NAVIO [8, 54] also showed significant but less pronounced reductions with a robotic outlier rate ranging from 5.3% to 5.8% (Table 3). Robotic precision was also evident in the application of personalised alignment strategies, as confirmed by postoperative full‐length lower limb CT‐scan [8]. Despite improved implant positioning precision, reviewed RCTs showed no statistically significant clinical advantages in PROMs, including WOMAC, OKS, KSS or HSS scores, regardless of alignment strategy (Table 4). For Clement et al. [13] although the overall WOMAC score differences were not statistically significant, the WOMAC pain component improved significantly in the RA‐TKA group (*p* = 0.029) at 2, 6, and 12 months postoperatively, considered clinically meaningful by the authors. The FJS, particularly sensitive to joint awareness, failed to show a significant difference between RA‐TKA and C‐TKA [13]. Nevertheless, subtle but meaningful improvements in quality‐of‐life domains such as vitality and emotional roles reported at two years suggest potential benefits warranting further investigation of modern patient‐specific outcomes with long‐term follow‐up [13, 33, 60]. Evidence from lower quality comparative and retrospective studies involving larger cohorts suggests that personalised alignment strategies in RA‐TKA may confer superior early PROMs and functional outcomes relative to C‐TKA [10, 11, 12, 20, 30, 52, 61]. Registry data have reported reduced revision rates, fewer manipulations under anaesthesia, lower systemic complication rates, and decreased postoperative opioid requirements for RA‐TKA compared to C‐TKA, reflecting potential advantages beyond alignment accuracy [45,]. However, these long‐term clinical benefits have yet to be confirmed in rigorous RCTs. These observations point toward potential clinical advantages of RA‐TKA in routine practice that may not be fully reflected in the more controlled environment of RCTs. Postoperative ROM between RA‐TKA and C‐TKA showed no clinically significant differences (Table 4) [33, 47, 49, 51]. Even the use of personalised alignment strategies in RA‐TKA [8] has not translated into superior postoperative ROM compared to mechanically aligned C‐TKA [19], reinforcing the limited impact of alignment strategy and implant accuracy alone on functional outcomes. Factors like pain control, rehabilitation protocols, soft‐tissue balancing, and prosthetic design may play a critical role. Van de Graaf et al. [58] have compared alignment strategies in RA‐TKA, finding that neither MA nor KA strategies alone consistently yielded balanced knees. However, FA achieved improved soft‐tissue balance across different knee phenotype subgroups [58]. These findings show the capacity of robotic systems to deliver precise and individualised alignment, allowing alignment strategies to be adapted to the specific bony anatomy and soft‐tissue envelope of each patient [60]. Conversely, the definition and execution of personalised alignment, including FA as one of its approaches, remain highly dependent on the intraoperative judgement of the surgeon regarding acceptable joint laxity in flexion and extension, as well as on the constraints and characteristics of the prosthetic design employed (e.g, PS or CR) [8, 13, 19, 26]. An increasing number of RCTs comparing RA‐TKA to C‐TKA are investigating personalised alignment strategies, reflecting continued progress [15, 27]. No workflow of the RCTs included evaluated intraoperative optimisation of the patellofemoral space, despite emerging strategies capable of intraoperative patellofemoral tracking assessment in RA‐TKA [28, 29, 44]. This represents a limitation in current literature, given the clinical significance of patellofemoral optimisation on TKA functional outcomes. In summary, RA‐TKA workflows have evolved substantially over the last two decades, transitioning from purely bone‐cutting technology [33, 47, 49, 51, 54] to decision‐making platforms capable of dynamic soft‐tissue assessment and alignment personalisation [8, 13, 19, 26]. Since approximately 2021, RCTs have increasingly adopted personalised alignment strategies and workflows guided by intraoperative robotic feedback, minimising the need for soft‐tissue release, although conventional MA C‐TKA represents the benchmark for comparison in all RCTs reviewed (Table 5).

RA‐TKA has demonstrated safety in managing complex deformities, retained hardware, and extra‐articular deformities, clinical scenarios where conventional instrumentation may be limited [31, 36, 40, 52]. However, evidence supporting its superiority in challenging cases, such as those involving obese patients remains uncertain [40]. Within the first postoperative week, RA‐TKA has also demonstrated transient but significant reductions in systemic and local inflammatory markers (IL‐6, IL‐8, TNF‐α, ESR and CRP), alongside reduced periarticular soft‐tissue and bone trauma, lower pain scores, and decreased opioid consumption compared to conventional techniques [19, 26]. Although these biological advantages are promising, they did not translate into lasting functional improvements or enhanced FJS, and should be weighed against potential risks of RA‐TKA, such as iatrogenic injury from pin placement for robotic frame installation [31], although none of the reviewed RCTs reported complications directly related to pin placement. There is increasing evidence supporting robotic assistance in unicompartmental knee arthroplasty (UKA), demonstrating improved implant positioning, fewer revisions, and faster recovery [4, 6, 25]. Given its precision, robotic assistance may enhance the precision and confidence of performing UKAs, potentially expanding patient eligibility for this procedure. Future trials should state UKA and TKA allocation criteria to fully evaluate robotic utility across the knee osteoarthritis spectrum [21]. Robotic technology, when considered within the continuum of progressive osteoarthritic changes and the 3D phenotypic spectrum of the knee, should offer surgeons the ability to confidently adapt the most appropriate surgical intervention, whether UKA or TKA, and select the optimal alignment strategy, be it kinematic, functional, or mechanical.

In the current scenario of robotic knee arthroplasty, there is considerable heterogeneity in system design, preoperative imaging requirements and intraoperative variability in surgeon evaluation of soft‐tissues and knee kinematics [39], highlighting the lack of definitive evidence favouring any specific robotic platform or surgical workflow [37, 53, 59]. Clinical utility of robotic assistance appears highly surgeon‐ and team‐dependent, suggesting robotic systems should be adopted based on surgeon proficiency and institutional resources, rather than assumed universal benefit [58]. Despite this evolution in robotic application, standardised reporting of surgical experience and learning curves remains inadequate. The learning curve was addressed in only three out of nine RCTs [47, 49, 54], all comparing TKA performed with MA. The average case numbers in the included RCTs (mean 69.7 ± 38) suggest that patients could have been enroled during or shortly after the learning curve. There is general consensus in the literature that a learning phase of approximately 6–36 cases is required to achieve proficiency in RA‐TKA, emphasising the importance of team coordination and procedural efficiency beyond alignment precision and operative time alone [57, 59]. Given the evolving use of robotic assistance into tools highly dependent on surgeon preference, enabling alignment customisation and dynamic soft‐tissue assessment, the learning curve appears increasingly critical, especially with personalised alignment strategies. However, recent RCTs seem to have moved beyond this debate, possibly underestimating the impact of the learning curve, while also demonstrating increased safety and advancements in robotic technology over the years [8, 13, 19, 26]. Nevertheless, comprehensive documentation of robotic experience, operative duration, and preoperative planning times should become standard practice in future trials to distinguish robotic‐specific outcomes from those attributable to surgeon proficiency with robotic assistance in TKA. The RACER trial [27] has further emphasised the importance of controlling surgeon experience, alignment strategy, and implant selection to fairly assess robotic system performance in real‐world settings, distinguishing inherent technology benefits from surgeon variability. From a cost‐effectiveness perspective, RA‐TKA in high‐volume surgeon settings has shown higher procedural costs than C‐TKA, without significant differences in length of stay or complications, although reporting reduced early readmissions rates [56]. Long‐term studies are required to ascertain if higher initial costs are offset by fewer revisions or improved functional outcomes. Robotic systems should be regarded as precision tools, with their clinical value closely tied to patient selection, surgeon and team experience, and institutional resources.

Some limitations should be considered in this review. The assessment of functional outcomes (e.g., WOMAC, OKS, KSS and ROM) was limited by heterogeneity in follow‐up durations and reporting among studies, making direct comparisons challenging. While some studies showed slight clinical superiority in the RA‐TKA group, these differences were generally small and not statistically significant. The subgroup comparison of robotic systems was also constrained by available data: all studies reporting functional outcomes involved either ROBODOC or Mako systems, with no trials involving imageless robotic systems, limiting conclusions on clinical differences between image‐based and imageless platforms. The exclusive focus on RCTs, though providing high‐quality evidence, may not fully reflect the variability and complexity of real‐world arthroplasty populations, including patients of wide age distribution, obesity, or valgus deformities. Overall, there is considerable heterogeneity among studies regarding robotic platforms, alignment strategies, soft‐tissue balancing techniques, and patellar management. Furthermore, the follow‐up durations were mostly short‐term, averaging less than two years, limiting insights into mid‐ and long‐term outcomes, such as implant survivorship.

## CONCLUSIONS

Over the past two decades, RA‐TKA has evolved substantially, transitioning from purely bone‐cutting technology aimed at MA to decision‐making platforms capable of alignment personalisation and dynamic soft‐tissue assessment, improving surgical precision regardless of the alignment strategy. Clinical superiority over C‐TKA remains uncertain in relatively low‐risk patients, predominantly older females with mild to moderate varus deformity. While early benefits, such as reduced pain, soft‐tissue injury and bone trauma, highlight the potential of RA‐TKA, reporting on long‐term outcomes is needed. Future research should focus on more heterogeneous patient populations, varied implant designs (e.g., medial‐stabilised), and stratification by 3D knee phenotype subgroups. Standardised reporting of learning curves, the use of modern outcome measures, and the integration of patellofemoral space optimisation will be essential. Addressing current methodological limitations and reflecting real‐world complexity will be critical to defining the future role of robotics in knee arthroplasty.

## AUTHOR CONTRIBUTIONS

All authors contributed to the study conception and design. Material preparation, data collection, and analysis were performed by Riccardo Sacco and Andrea Tecame. The first draft of the manuscript was written by Riccardo Sacco, and all authors commented on previous versions of the manuscript. All authors read and approved the final manuscript.

## CONFLICT OF INTEREST STATEMENT

The authors declare no conflicts of interest.

## ETHICS STATEMENT

None declared.

## Supporting information

PRISMA checklist.

PRISMA_protcol.

**Table 1 Supplementary Materials.** The systematic literature search was conducted acrossPubMed, the Cochrane Library, and Google Scholar, covering the period from January 1, 2000, to January 1, 2025. Boolean search strategies were applied in each database, focusing on terms related to total knee arthroplasty, robotic assistance, and conventional surgical techniques. The comprehensive search yielded a total of 850 records: 500 from PubMed, 113 from the Cochrane Library, and 237 from Google Scholar. **Table 2 Supplementary Materials**. Only Level 1 randomized controlled trials (RCTs) published in English between January 1, 2000, and January 1, 2025, were included. Studies were excluded if they lacked a comparative group, were not available in full‐text format, were not RCTs, and did not provide an explicit report on the surgical workflow. This approach aligns with methodologies used in previous systematic reviews on robotic‐assisted TKA. **Table 3 Supplementary Materials**. **PICO Framework for Systematic Review.** This PICO framework was used to guide the eligibility criteria, search strategy, and data extraction in this PRISMA‐compliant systematic review. It supports a focused synthesis of Level I RCTs evaluating the evolving role of RA‐TKA compared to C‐TKA, particularly in the context of alignment strategies, surgical workflows, and outcome measures over the past two decades.

## Data Availability

The data supporting the findings of this study are available from the corresponding author upon reasonable request.
