# Supplementary material for: Robotic vs. conventional total knee arthroplasty over two decades: Evolving trends toward personalised alignment without significant clinical superiority in predominantly mild varus deformity—A systematic review of RCTs
Source: J Exp Orthop. 2025 Dec 2;12(4):e70452. doi: 10.1002/jeo2.70452 (PMC12670295; doi:10.1002/jeo2.70452)
Supplement: Supplementary file 2 — PRISMA_protcol. [file JEO2-12-e70452-s002.docx]

**PRISMA Protocol.**

**Title**

Robotic versus Conventional Total Knee Arthroplasty: Trends in Surgical Workflows, Alignment strategies, and Outcomes measurements over the Past Two Decades. A Systematic Review of Randomized Controlled Trials.

**1. Rationale**

Robotic-assisted total knee arthroplasty (RA-TKA) has emerged as a promising advancement aimed at enhancing surgical precision, component alignment, and patient outcomes in knee replacement surgery. However, randomized controlled trials (RCTs) comparing RA-TKA and conventional total knee arthroplasty (C-TKA) have revealed substantial variability in alignment strategies, surgical workflows, and outcome measures based on surgeon experience and preferences. These differences reflect both the heterogeneity of robotic platforms and the evolving trends in knee arthroplasty techniques. A comprehensive synthesis of high-quality RCTs is essential to evaluate the comparative effectiveness of RA-TKA, clarify its clinical value, and understand how its role has developed in light of technological innovation and changing surgical paradigms, over the past two decades.

**2. Objectives**

To systematically review Level I RCTs comparing RA-TKA and C-TKA with respect to surgical workflow, alignment techniques, functional outcomes, radiographic alignment, soft-tissue handling, and learning curve over the last two decades (2000-2024).

**3. Eligibility Criteria**

**Inclusion Criteria**

- Randomized controlled trials (Level I evidence)
- Adult patients undergoing primary TKA for end-stage primary osteoarthritis
- Comparison between robotic-assisted TKA and conventional/manual TKA
- Reports on surgical workflow: planning, bone resection, alignment strategy (mechanical/kinematic/functional), gap balancing, soft-tissue handling
- English-language full-text publications
- Minimum follow-up: 1 month
- Clinical and/or radiographic outcomes reported (e.g., PROMs, ROM, alignment outliers)
- Use of commercially available robotic systems and standard implant designs
- Studies with clear demographic data (age, sex, BMI)

**Exclusion Criteria**

- Non-RCTs, case series, reviews
- Inflammatory or post-traumatic arthritis
- Revision or unicompartmental knee arthroplasty
- Custom or investigational implants
- Less than 1-month follow-up
- No extractable outcome or demographic data
- Non-English publications without translation
- Studies lacking comparative analysis or workflow description

**4. Information Sources**

- **Databases:** PubMed, Cochrane Library, Google Scholar
- **Search Dates:** January 1, 2000 – January 1, 2025
- **Manual Search:** Reference lists of included studies

**5. Search Strategy**

Boolean strategy adapted to each database including the following keywords and their variations: “Knee Arthroplasty,” “Knee Replacement,” “Joint Replacement,” “Total Knee,” “Robotic Assisted,” “Robotic Surgery,” “Robotic,” “Conventional,” “Manual,” and “Standard Technique”

**6. Study Selection Process**

- Two reviewers (RS, AT) will independently screen all titles/abstracts and full texts
- Records will be managed in EndNote; duplicates removed
- Discrepancies will be resolved by a third senior reviewer (PA)
- Reference lists will bescreened for additional eligible studies

**7. Data Extraction and Items**

A standardized Excel spreadsheet will be used to extract:

- Author, year
- Study design and sample size
- Demographics: age, sex, BMI
- Robotic system and implant type
- Surgical workflow details
- Alignment strategy
- Outcomes:
  - Radiographic alignment
  - PROMs (WOMAC, OKS, KSS, HSS)
  - ROM
  - FJS
  - Inflammatory markers, tissue injury
  - Learning curve and operative time
  - Complications
- Follow-up duration

**8. Data Synthesis**

- Qualitative synthesis only (due to expected heterogeneity in robotic platforms and surgical workflows)
- Descriptive statistics (means, SDs, ranges)
- No meta-analysis planned
- Results categorized chronologically and by robotic system/workflow/alignment strategy

**9. Amendments**

Any future protocol amendments will be documented with rationale and dates.

**10. Funding and Conflicts of Interest**

No funding will be received for this review. Authors declare no conflicts of interest.
