# Supplementary material for: Robotic vs. conventional total knee arthroplasty over two decades: Evolving trends toward personalised alignment without significant clinical superiority in predominantly mild varus deformity—A systematic review of RCTs
Source: J Exp Orthop. 2025 Dec 2;12(4):e70452. doi: 10.1002/jeo2.70452 (PMC12670295; doi:10.1002/jeo2.70452)
Supplement: Supplementary file 3 — Table 1 Supplementary Materials. The systematic literature search was conducted acrossPubMed, the Cochrane Library, and Google Scholar, covering the period from January 1, 2000, to January 1, 2025. Boolean search strategies were applied in each database, focusing on terms related to total knee arthroplasty, robotic assistance, and conventional surgical techniques. The comprehensive search yielded a total of 850 records: 500 from PubMed, 113 from the Cochrane Library, and 237 from Google Scholar. Table 2 Supplementary Materials. Only Level 1 randomized controlled trials (RCTs) published in English between January 1, 2000, and January 1, 2025, were included. Studies were excluded if they lacked a comparative group, were not available in full‐text format, were not RCTs, and did not provide an explicit report on the surgical workflow. This approach aligns with methodologies used in previous systematic reviews on robotic‐assisted TKA. Table 3 Supplementary Materials. PICO Framework for Systematic Review. This PICO framework was used to guide the eligibility criteria, search strategy, and data extraction in this PRISMA‐compliant systematic review. It supports a focused synthesis of Level I RCTs evaluating the evolving role of RA‐TKA compared to C‐TKA, particularly in the context of alignment strategies, surgical workflows, and outcome measures over the past two decades. [file JEO2-12-e70452-s003.docx]

**Table 1 Supplementary Materials.** The systematic literature search was conducted across PubMed, the Cochrane Library, and Google Scholar, covering the period from January 1, 2000, to January 1, 2025. Boolean search strategies were applied in each database, focusing on terms related to total knee arthroplasty, robotic assistance, and conventional surgical techniques. The comprehensive search yielded a total of 850 records: 500 from PubMed, 113 from the Cochrane Library, and 237 from Google Scholar.

| **Database** | **Search Strategy** |  |
| --- | --- | --- |
| **PubMed** | ("Knee Arthroplasty" OR "Knee Replacement" OR "Joint Replacement" OR "Total Knee") AND ("Robotic Assisted" OR "Robotic Surgery" OR "Robotic") AND ("Conventional" OR "Manual" OR "Standard Technique") | 500 |
| **Cochrane Library** | ("Knee Arthroplasty" OR "Knee Replacement" OR "Joint Replacement" OR "Total Knee") AND ("Robotic Assisted" OR "Robotic Surgery" OR "Robotic") AND ("Conventional" OR "Traditional" OR "Standard Technique") | 113 |
| **Google Scholar** | ("Knee Arthroplasty" AND "Knee Replacement" AND "Joint Replacement" AND "Total Knee") AND ("Robotic Assisted" AND "Robotic Surgery" AND "Robotic") AND ("Conventional" AND "Manual" OR "Standard Technique") | 237 |
| **Total** |  | 850 |

**Table 2 Supplementary Materials**. Only Level 1 randomized controlled trials (RCTs) published in English between January 1, 2000, and January 1, 2025, were included. Studies were excluded if they lacked a comparative group, were not available in full-text format, were not RCTs, and did not provide an explicit report on the surgical workflow. This approach aligns with methodologies used in previous systematic reviews on robotic-assisted TKA.

| **Inclusion criteria** | **Exclusion criteria** |
| --- | --- |
| Randomized controlled trials (RCTs) with Level I evidence comparing robotic-assisted TKA (RA-TKA) to conventional TKA (C-TKA).  Adults undergoing primary TKA for end-stage primary osteoarthritis.  Studies explicitly reporting surgical workflow elements: preoperative planning, intraoperative bone resections, alignment strategy (mechanical, kinematic or functional), gap balancing techniques, and soft-tissue management.  Studies using commercially available robotic systems with defined implant designs.  Minimum follow-up duration of 1 month postoperatively with documented clinical or radiographic outcomes.  English-language full-text articles with accessible and extractable data.  Studies providing comparative outcome metrics, including patient-reported outcomes (e.g., WOMAC, OKS, HSS, KSS), range of motion (ROM), mechanical alignment parameters.  Studies with clearly defined patient demographics (age, sex, BMI). | Non-RCTs studies (e.g., retrospective, cross-sectional, cohort studies), reviews, case series.  Studies involving patients with inflammatory arthritis (e.g., rheumatoid arthritis), post-traumatic arthritis, or other non-degenerative etiologies.  Patients undergoing revision TKA or UKA.  Use of custom implants or investigational devices not widely available.  Studies with <1 month follow-up or insufficient outcome data.  Non-English language publications without available translation.  Studies not reporting relevant clinical or radiological outcome measures or lacking disaggregated data by intervention group.  Studies with incomplete or missing demographic data. |

**Table 3 Supplementary Materials**. **PICO Framework for Systematic Review.** This PICO framework was used to guide the eligibility criteria, search strategy, and data extraction in this PRISMA-compliant systematic review. It supports a focused synthesis of Level I RCTs evaluating the evolving role of RA-TKA compared to C-TKA, particularly in the context of alignment strategies, surgical workflows, and outcome measures over the past two decades.

| Population (P) | Adults undergoing primary total knee arthroplasty (TKA) for end-stage osteoarthritis. |
| --- | --- |
| Intervention (I) | Robotic-assisted total knee arthroplasty (RA-TKA) using image-based or imageless systems. |
| Comparator (C) | Conventional/manual total knee arthroplasty (C-TKA) performed with standard instrumentation following mechanical alignment principles. |
| Outcomes (O) | Primary outcomes: adoption of alignment strategies and surgical workflows, alignment outliers, functional scores (WOMAC, HSS, KSS, OKS, FJS), knee range of motion, learning curve. Secondary outcomes: complications, inflammatory markers, and quality-of-life measures. |
| Study Design | Systematic review of Level 1 Randomized controlled trials (RCTs) published between 2000 and 2024. |
